# A Micropropagation Protocol for the Endangered Medicinal Tree *Prunus africana* (Hook f.) Kalkman: Genetic Fidelity and Physiological Parameter Assessment

**DOI:** 10.3389/fpls.2020.548003

**Published:** 2020-11-26

**Authors:** Richard Komakech, Yong-Goo Kim, Wook Jin Kim, Francis Omujal, Sungyu Yang, Byeong Cheol Moon, Denis Okello, Endang Rahmat, Grace Nambatya Kyeyune, Motlalepula Gilbert Matsabisa, Youngmin Kang

**Affiliations:** ^1^ Herbal Medicine Resources Research Center, Korea Institute of Oriental Medicine (KIOM), Naju-si, South Korea; ^2^ Korean Convergence Medicine Major KIOM, University of Science and Technology (UST), Daejeon, South Korea; ^3^ Natural Chemotherapeutics Research Institute (NCRI), Ministry of Health, Kampala, Uganda; ^4^ Gombe Secondary School, Mpigi, Uganda; ^5^ IKS Research Group, Department of Pharmacology, Faculty of Health Sciences, University of the Free State, Bloemfontein, South Africa

**Keywords:** chemical characterization, chlorophyll, genetic fidelity, medicinal plant, micropropagation, photosynthesis, Prunus africana

## Abstract

*Prunus africana* is an endangered medicinal plant and hence new propagation methods are urgently required to increase its populations. Unfortunately, propagation through seeds is challenging due to its long flowering cycle and recalcitrant seeds. We developed a protocol for micropropagation using nodal segment explants. A woody plant medium supplemented with vitamins, 15 g L^−1^ sucrose, and 1.0 mg L^−1^ 6-benzylaminopurine (BAP) supported the optimum rate (100%) of axillary shoot initiation. Supplementation with 15 g L^−1^ sucrose and 1.5 mg L^−1^ indole-3-acetic acid (IAA) provided the optimum rate (75%) of root initiation. Rooted plantlets were successfully planted in sterilized horticultural soil containing perlite (2:1 *v/v*) and the survival rate was 98% following acclimatization. The photosynthetic rate assessed using FlourPen FP110 series showed that the ratio of variable fluorescence to maximum fluorescence mean value for *in vitro* regenerated *P. africana* (0.830 ± 0.0008) was similar to that of the maternal *P. africana* plant (0.825 ± 0.005), indicating similarity in their photosynthetic performance; a pivotal process for growth and development. The Fourier transform near-IR (FT-NIR) spectrometer analysis of the *in vitro* regenerated and the maternal *P. africana* plant samples exhibited homogeneity in the absorbance peaks at 8,273, 6,344, and 4,938–4,500 cm^−1^ associated with lipids, starch, and proteins. The genetic fidelity of regenerated plants was confirmed using the randomly amplified polymorphic DNA (RAPD) technique. Our protocol is suitable for use in large-scale *P. africana* to meet the increasing demands for it in the global market.

## Introduction

Micropropagation of medicinal plants has become highly important in providing quality stock plants to meet conservation and pharmaceutical needs (Nilanthi and Yang, 2014). *Prunus africana* (Hook f.) Kalkman (Family Rosaceae); common name African Cherry is one of the most popular medicinal plants on the global market (Bodeker et al., 2014). It is native to Sub-Saharan Africa and has been used in traditional medicine since time immemorial (Jimu, 2011; Komakech et al., 2017). A study using six nuclear microsatellites showed that *P. africana* exhibits strong divergence among five main Afromontane regions of West Africa, East Africa west of the eastern rift valley, East Africa east of the eastern rift valley, southern Africa, and Madagascar; a variation that may be associated to pleistocene changes in climatic conditions (Kadu et al., 2013). Similarly, a study using six nuclear and five plastid microsatellites showed that although all plastid haplotypes found in Ethiopia belonged to one single lineage; the populations from East Africa and Madagascar contain haplotypes from up to four more divergent lineages (Mihretie et al., 2015). It is a unique medicinal plant, which is known for its therapeutic values for a number of diseases, including prostate cancer, benign prostatic hyperplasia, erectile dysfunction, urinary tract disorders, skin lacerations, kidney disease, chest pain, stomach upset, inflammation, and as an aphrodisiac (Steenkamp, 2003; Jimu, 2011; Ngule et al., 2014; Ochwang’i et al., 2014; Koros et al., 2016a; Komakech and Kang, 2019). The importance of *P. africana* in ethnomedicine has led to an increase in global demand, which has resulted in over-exploitation and concerns over conservation (Stewart, 2003; Koros et al., 2016b; Komakech and Kang, 2019). Consequently, in 1995, *P. africana* was listed in appendix II of the Convention on International Trade in Endangered Species of Wild Fauna and Flora (CITES) in an attempt to protect and conserve it (Cunningham et al., 2016). In Cameroon, *P. africana* is outlined as endangered in the IUCN checklist for non-detriment Findings (IUCN 2001; Betti, 2008). To date, various propagation methods have been explored in the attempt to replenish dwindling populations of *P. africana*, including the use of seeds and stem cuttings (Cunningham and Mbenkum, 1993; Tchoundjeu et al., 2002; Kebede et al., 2013). However, although the seeds of *P. africana* germinate when sown in favorable soil conditions (Cunningham et al., 2016), their germination rates reduce significantly within a short time after harvest (Sacandé et al., 2004). In fact, the seeds have been classified as recalcitrant in nature in that they lose viability with loss of humidity (Negash, 2004) or semi-recalcitrant (Nzweundji et al., 2019). The use of other *P. africana* propagation methods have been explored in recent years, including stem cuttings using a non-mist poly-propagator (Tchoundjeu et al., 2002; Kebede et al., 2013). Unfortunately, this conventional method of vegetative propagation is considerably limited by variations in biotic and abiotic environmental conditions (Min et al., 2010). Consequently, the use of micropropagation techniques may provide a better alternative and enable rapid multiplication of *P. africana* propagules. Indeed, micropropagation methods have been extensively applied for the rapid multiplication of many other medicinal plant species (Pospíšilová et al., 1999). This is the first study to develop a protocol for the efficient micropropagation of *P. africana* from juvenile nodal shoot segments obtained from 1-year-old *P. africana* plants. The explants were sterilized and the axillary shoots were induced, rooted, and acclimatized. However, considering that micropropagation of plants is sometimes associated with somaclonal variation (Ning et al., 2007; Goyal et al., 2015), the genetic fidelity of the *in vitro* regenerated *P. africana* plants was assessed using randomly amplified polymorphic DNA (RAPD). Furthermore, a Fourier transform near-IR (FT-NIR) spectrometer, soil-plant analysis development (SPAD), and FlourPen FP110 series OJIP protocol were used to compare the chemical compositions, chlorophyll pigments, and photosynthetic rate, respectively, of the maternal and *in vitro* regenerated *P. africana* plants.

## Materials and Methods

### Plant Material and Preparation of Explants

**Figure 1:**
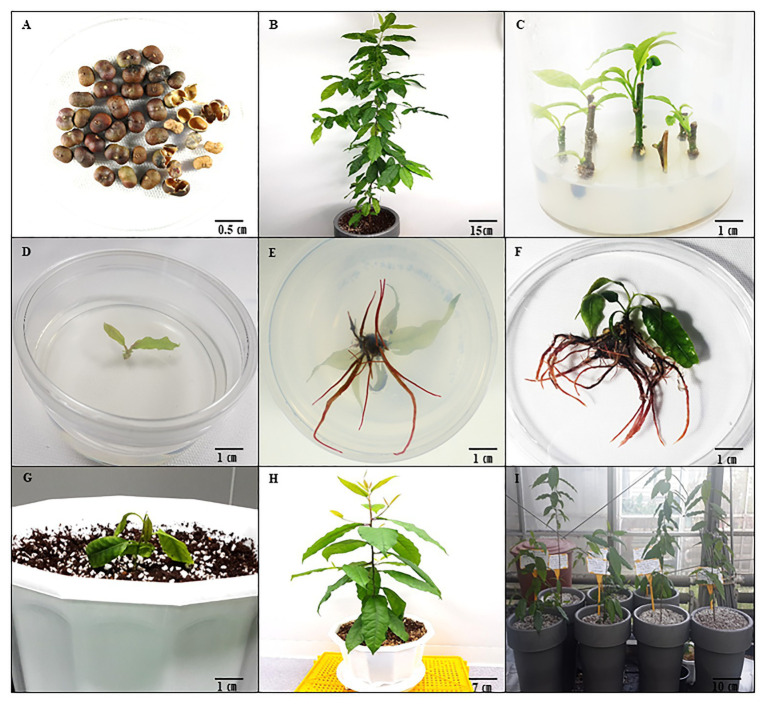
*Prunus africana in vitro* propagation through axillary shooting from nodal segments in optimum growth conditions. **(A)** Seeds of *P. africana*. **(B)** Maternal *P. africana* plant from germinated seeds. **(C)** Axillary shoots formed on the *P. africana* nodal segment cultured in Woody Plant Medium (WPM) with vitamins supplemented with 15 g L^−1^ sucrose and 1.0 mg L^−1^ 6-Benzylaminopurine (BAP). **(D,E)** Rooting of excised axillary shoots in WPM with vitamins supplemented with 15 g L^−1^ sucrose, and 1.5 mg/L^−1^ indole-3-acetic acid (IAA). **(F)** *In vitro* rooted *P. africana* plantlets. **(G)** *Prunus africana* plantlet planted in horticulture soil mixed with perlite in the ratio of 2:1. **(H)** Acclimatized regenerated *P. africana* plant with well-developed root and shoot systems in horticulture soil mixed with perlite in the ratio of 2:1. **(I)** Regenerated *P. africana* in greenhouse.

*Prunus africana* seeds (Figure 1A), collected from a single maternal plant in the medicinal garden at the Natural Chemotherapeutics Research Institute, Ministry of Health, Uganda, East Africa. A voucher specimen, number KYM-KIOM-201901, was deposited at the Korean Herbarium of Standard Herbal Resources (Index Herbarium code: KIOM) in the Korea Institute of Oriental Medicine (KIOM), Herbal Medicine Resources Research Center, Republic of South Korea. The seeds were germinated and, following 1 year of growth in the greenhouse (Figure 1B), juvenile shoots with nodal buds were collected. Shoots were washed under a continuous flow of tap water for 30 min. The shoots were then cut into short pieces (30–40 mm in length) and thoroughly washed with autoclaved double-distilled water. The washed nodal segments were then surface sterilized with 70% (v/v) ethanol (Daihan Scientific, Siheung, Korea) for 4 min before being thoroughly rinsed three times with autoclaved double-distilled water. Furthermore, they were surface sterilized with 3% sodium hypochlorite [prepared by diluting 12% sodium hypochlorite (Yakuri Pure Chemicals Co., Ltd., Kyoto, Japan)] for 3 min and rinsed three times with autoclaved double-distilled water to remove all traces of the disinfectant. The sterilized shoots were cut into nodal segments (15–20 mm) using a sterile scalpel. All sterilization processes were conducted on a laminar flow clean bench.

All media and growth regulators used in this study were purchased from Duchefa Biochemie B. V., Haarlem, Netherlands. In set-up 2.2, 2.3, 2.4, and 2.5, the PH of the medium was adjusted to 5.8 by adding a few drops of 1 N hydrochloric acid or sodium hydroxide before autoclaving at 121°C and all the steps were repeated three times to ensure its reproducibility. The experimental set-up were maintained under white fluorescent light at 33.73 μmol m^−2^ s^−1^ intensity with a 16 h light and 8 h dark cycle in a laboratory at 25 ± 1°C.

### Culture Media Effects on Axillary Shoot Initiation and Growth

Six plant growth basal media were screened for their effects on *P. africana* nodal segment axillary shoot initiation and growth. Stem nodal segments (15–20 mm) were inoculated vertically in 100 ml of each of the six solid basal media; Murashige and Skoog (MS; Murashige and Skoog, 1962), Woody Plant Medium (WPM; Lloyd and MCCown, 1980), Gamborg (B5; Gamborg, et al., 1968), Quoirin and Lepoivre (QL; Quoirin and Lepoivre, 1977), Linsmaier and Skoog (LS; Linsmaier and Skoog, 1975), and De Greef and Jacobs (DJ; De Greef and Jacobs, 1979). The experiment was done in a polystyrene culture vessel (125 × 110 Gaooze 1011C culture vessel, Gyeonggi-do, Korea). Each basal medium contained vitamins and was supplemented with 30 g L^−1^ sucrose before being gelled in 8 g L^−1^ of phyto agar (Duchefa Biochemie 2003 RV, Haarlem, Netherlands). Twenty-four nodal segment explants (8 explants per culture vessel × 3 replicates) were inoculated in each medium. The axillary shoot initiation rate and the length of axillary shoots formed were measured and recorded after 6 weeks.

### Sucrose Effects on Axillary Shoot Initiation and Growth

The effects of various concentrations of sucrose at 0, 15, 30, 45, 60, 75, 90, and 105 g L^−1^ on the growth of axillary buds in *P. africana in vitro* were investigated. Stem nodal segments (15–20 mm long) were vertically sub-cultured in 100 ml WPM (chosen as the optimum medium), with vitamins and was supplemented with different sucrose concentrations gelled in 8 g L^−1^ phyto agar in a polystyrene culture vessel (125 × 110 Gaooze 1011C culture vessel, Gyeonggi-do, Korea). Twenty-four nodal segments (8 explants per culture vessel × 3 replicates) were used in each treatment. The axillary shoot initiation rate and the length of axillary shoots formed were measured and recorded after 6 weeks.

### Cytokinin Effects on Axillary Shoot Initiation and Growth

Stem nodal segments (15–20 mm long) were sub-cultured in 100 ml WPM containing vitamins and 15 g L^−1^ sucrose (optimum sucrose condition) and supplemented with different cytokinins, 6-Benzylaminopurine (BAP), Thidiazuron (TDZ), Kinetin (KN), isopentenyl adenine (2iP), and Zeatin, at varying concentrations of 0.5, 1.0, 1.5, and 2.0 mg L^−1^ and gelled in 8 g L^−1^ of phyto agar in a polystyrene culture vessel (125 × 110 Gaooze 1011C culture vessel, Gyeonggi-do, Korea). In each treatment, 24 nodal shoot segments (8 explants per culture vessel × 3 replicates) were used. After 6 weeks, the axillary shoot initiation rate and length of axillary shoots formed in each treatment were recorded.

### Root Initiation and Growth

Axillary shoots (10–15 mm long) excised from sub-cultured nodal segments (Figures 1C,D) were transferred to 30 ml WPM containing vitamins and 15 g L^−1^ of sucrose supplemented with different auxins, indole-3-butyric acid (IBA), naphthaleneacetic acid (NAA), and indole-3-acetic acid (IAA), at 0.5, 1.0, 1.5, and 2.0 mg L^−1^ concentrations and gelled in 8 g L^−1^ of phyto agar in a polystyrene plant culture dish (100 × 40 SPL Life sciences 310,100 Plant culture dish, Gyeonggi-do, Korea). For each treatment, 24 excised axillary shoots (1 explant per petri dish × 24 replicates) were used. After 6 weeks, rooting rates (percentage), root numbers, and root lengths were recorded for each treatment.

### Acclimatization

*Prunus africana* plantlets with well-developed roots after 6 weeks were collected from the culture dishes, and the roots were washed with sterile distilled water to remove the adhered culture media. They were then transferred into plastic pots (22 cm in diameter) containing sterile horticultural soil mixed with perlite in the ratio of 2:1 and maintained in a greenhouse. The survival rate was then determined after 2 months of growth in the greenhouse.

### Isolation of Genomic DNA and RAPD Analysis

To confirm the genetic fidelity of the *in vitro* regenerated plants, RAPD analysis was conducted on 6-month-old greenhouse *in vitro* regenerated and maternal (control) *P. africana* plants. Genomic DNA was extracted from 100 mg of fresh leaf tissue obtained from randomly picked leaves from both the maternal plant and the *in vitro* regenerated plants with a DNeasy Plant Mini Kit (Qiagen, Germany). Purified DNA was stored at −20°C before further analysis. RAPD amplification was performed in a reaction volume of 30 μl containing a 10 ng DNA template, 10 pmol of each random primer, and a Solg™ 2X Taq PCR Smart-Mix I (Solgent, Daejeon, Korea). The amplification cycle consisted of an initial denaturation step at 95°C for 5 min, followed by 35 cycles of 30 s at 95°C, 30 s at 46°C, and 45 s at 72°C, with a final extension step of 7 min at 72°C. The amplification products were separated using a 1.5% agarose gel containing Eco Dye (Biofact, Daejeon, Korea). The sizes of the amplification products were determined by comparison with a 1 kb DNA ladder (Solgent, Daejeon, Korea). The DNA bands in the gel were visualized under the Gel Doc System (Syngene, Cambridge, United Kingdom) for photography and digitalization of images.

### Fourier Transform Near-IR Analysis

The leaves (001), stems (003), and roots (005) of regenerated plants and the leaves (002), stems (004), and roots (006) of the maternal plant (grown under the same conditions in the greenhouse) were collected and dried in an oven at 60°C. Each leaf, stem, and root sample was ground using a steel pulverizing machine (250G New Type Pulverizing Machine, Model RT-N04-2V, Taiwan) at 25,000 rpm to provide a total of six samples. Three grams of each of the finely powdered samples were placed in 22 mm vials and analyzed using a TANGO FT-NIR spectrometer (Bruker Optics, Billerica, MA, United States). Prior to the analysis, the TANGO FT-NIR spectroscopy was first calibrated using a Light Trap (Type 1002961, ECL 00) and Gold standard (Type 1024957, ECL:01). The spectra, formed over wave numbers 12,500–3,950 cm^−1^, were determined based on the different functional groups in the samples. The data obtained were standardized with noise reduction and a dendrogram for each sample was constructed based on Ward’s algorithm clustering with characteristic data pre-processing first derivative and vector normalization, and a standard Euclidean distance and frequency range of 9,981–4,014 cm^−1^. OPUS TANGO-R software was used for the Ward algorithm. The minimum variance method for cluster analysis was used, and the homogeneous groups were sorted to the maximum extent possible.

### SPAD Chlorophyll Content Measurement

**Figure 2:**
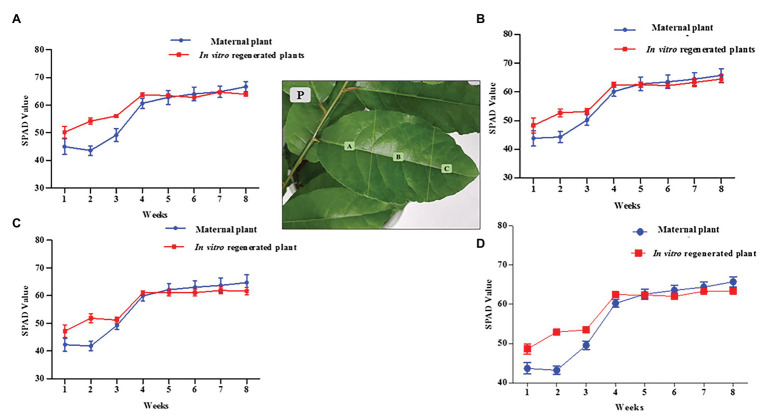
Soil-plant analysis development (SPAD) chlorophyll content measurement for *in vitro* regenerated and maternal *P. africana* plants. **(P)** Leaf surface regions in which chlorophyll was measured. **(A)** SPAD value for chlorophyll contents at base region of the leaves. **(B)** SPAD value for chlorophyll contents at mid leaves region. **(C)** SPAD value for chlorophyll contents at apex region of the leaves. **(D)** Overall total average SPAD value for chlorophyll measured across the leaves surfaces.

The chlorophyll pigments of the 6 months old *in vitro* regenerated plants (grown under the same conditions as the maternal plant of about 2 years in the greenhouse) were determined using a SPAD chlorophyll meter (Minolta Camera, Co., Japan). The calibrated SPAD meter was carefully clamped over a *P. africana* leaf to obtain the chlorophyll readings. The first six leaves on each plant from the terminal bud were chosen due to their longevity over the duration of the experiment. SPAD readings were taken at weekly intervals on the same leaves for a duration of 8 weeks. We calculated each leaf SPAD value as the average of four readings taken at the leaf base region (A), the mid leaf region (B), and the leaf apex region (C; Figure 2P). The overall chlorophyll pigment distribution across a leaf surface was then obtained by adding the average values from the different regions (A + B + C). Four replicates each (24 leaves) for *in vitro* regenerated and maternal *P. africana* treatments were used to obtain the overall SPAD chlorophyll average values once a week for 8 weeks.

### Measurement of Chlorophyll Fluorescence

Leaf clip gaskets were used to dark-adapt the leaves for 40 min prior to measurements of the chlorophyll fluorescence using a FlourPen FP110 series (Spol. Sr.o. Drasov 470, 664 24 Drasov, Czech Republic). Measurements were taken once per week at the same time every day (14:00) for 8 weeks. The FlourPen FP110 series OJIP protocol was used to obtain the Fv/Fm values using a saturating flash of over 4,000 μmol m^−2^ s^−1^. The Fv, variable chlorophyll fluorescence (Fm-Fo), was measured in the dark-adapted state, when non-photochemical processes are minimal. The Fo, minimal chlorophyll fluorescence intensity, was measured in the dark-adapted state when all photosystem II (PSII) reaction centers are open. The Fm, maximal chlorophyll fluorescence intensity, was measured in the dark-adapted state during the application of the saturation pulse of light. Five replicates each of the regenerated and the maternal *P. africana* plants were used.

### Data Analysis

The following formulae were used to calculate the percentage of certain parameters:

Axillary shoot initiation rate = (*N/M*)100. Where *N* is number of nodal segments inoculated in a growth medium that formed axillary shoots at 6 weeks and *M* is the total number of nodal segments inoculated in the growth medium.

Rooting rate = (*P/Q*)100. Where *P* is the number of inoculated shoots in a growth medium that formed roots at 6 weeks and *Q* is total number of inoculated shoots.

Survival rate = (*R/S*)100. Where *R* is the number of plantlets that survived at 2 months and *S* is the total initial number of plantlets transferred to sterile horticulture soil mixed with perlite in the ratio of 2:1 in plastic pots.

The data were analyzed using a one-way ANOVA, and means were separated using Bonferroni’s *post hoc* test using Graph Pad Prism (Graph Pad software, Ver. 5.03) and FT-IR spectroscopy multivariate statistical analysis. OPUS TANGO-R software was used to calculate the Ward algorithm using the minimum variance method for cluster analysis where homogeneous groups were sorted to the maximum extent possible.

Each value represents the mean ± SE of the replicates.

## Results

### Media Effects on Axillary Shoot Growth

**Figure 3:**
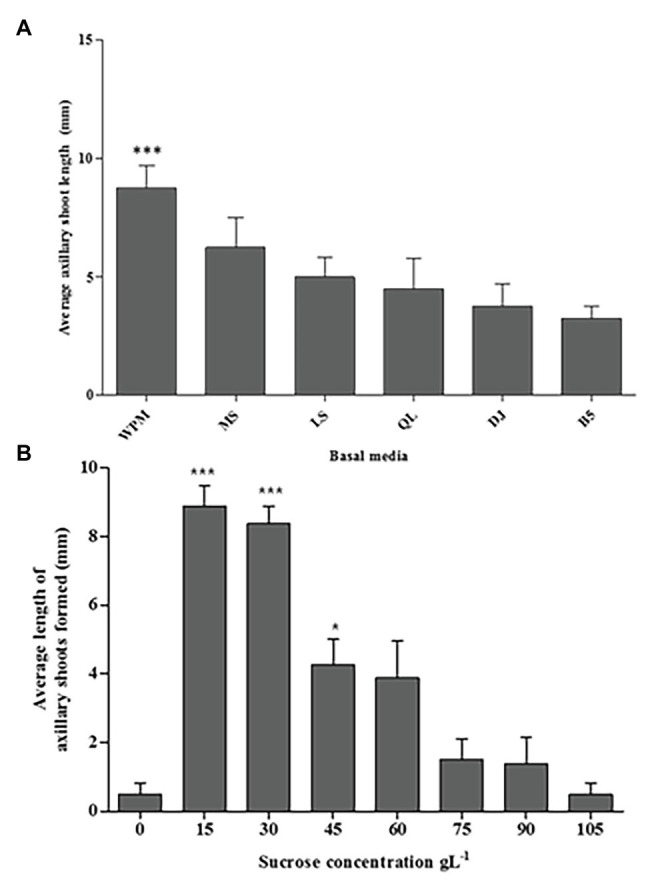
The media and sucrose effects on *in vitro P. africana* axillary shoots initiation and growth from the nodal segments. **(A)** The average length of the axillary shoots in different media, including vitamins supplemented with 30 g L^−1^ sucrose at 6 weeks of culture. **(B)** The average length of the axillary shoots in different sucrose concentrations. The indicated statistical differences are calculated using the one-way ANOVA and Bonferroni’s *post hoc* test. ^***^ *p* < 0.0001 and ^*^ *p* < 0.05.

In this study, all media (WPM, MS, LS, QL, DJ, and B5 supplemented with 30 g L^−1^ sucrose) enhanced axillary shoot initiation from the nodal segments of *P. africana* after 6 weeks of *in vitro* culture (Figure 3A). The mean value for axillary shoot lengths obtained from nodal segments cultured in WPM (9.30 ± 0.53 mm) was observed to be significantly high (*p* < 0.0001) compared to that from other media with the lowest mean axillary shoot length (3.30 ± 0.29 mm) recorded in the B5 medium after 6 weeks. Consequently, WPM supported the optimum axillary shoot growth in this experiment.

### Sucrose Effects on Axillary Shoot Initiation and Growth

Axillary shoot outgrowth was found to be 100% in nodal segments cultured in the WPM containing vitamins supplemented with 15 or 30 g L^−1^ of sucrose compared to only 25% axillary shoot formation in the WPM containing vitamins supplemented with 0 or 105 g L^−1^ of sucrose. Nodal segments cultured in medium with 15 or 30 g L^−1^ of sucrose had significant (*p* < 0.0001) axillary shoot length formed compared to the other treatments with the highest mean axillary shoot length (8.88 ± 0.680 mm) recorded in nodal segments cultured in WPM containing vitamins supplemented with 15 g L^−1^ of sucrose, followed by nodal segments cultured under the same conditions but supplemented with 30 g L^−1^ of sucrose (mean shoot length = 8.38 ± 0.240 mm; Figure 3B). Increasing concentrations of sucrose in the growth media tended to decrease the development and growth of axillary shoots. The lowest axillary shoot mean lengths (0.50 ± 0.260 mm) was recorded in WPM containing vitamins supplemented with 105 g L^−1^ of sucrose and in the control treatment where no sucrose was added to the growth medium. From these results, 15 g L^−1^ of sucrose gave the optimum condition for the *P. africana* axillary shoot growth.

### Cytokinin Effects on Axillary Shoot Growth

**Figure 4:**
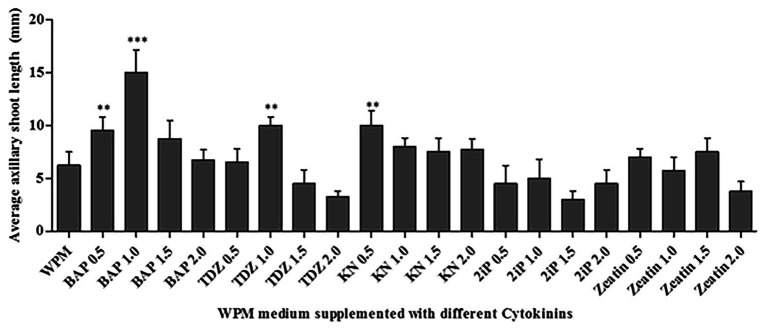
Average axillary shoot length of *P. africana* from nodal explants cultured in WPM with vitamins supplemented with 15 g L^−1^ sucrose and different cytokinins-BAP, Thidiazuron (TDZ), Kinetin (KN), isopentenyl adenine (2iP), and Zeatin at different concentrations at 6 weeks. The indicated statistical differences are calculated using the one-way ANOVA and Bonferroni’s *post hoc* test. ^**^ *p* < 0.001; and ^***^ *p* < 0.0001.

All cytokinins used (BAP, TDZ, KN, 2iP, and Zeatin) at all concentrations tested promoted shoot growth of at least 2.5 mm length on average in the WPM containing vitamins supplemented with 15 g L^−1^ of sugar, after 6 weeks (Figure 4). However, the shoot growth varied depending on the cytokinin type and concentration. The statistical analysis showed that 1.0 mg L^−1^ of BAP resulted in significantly (*p* < 0.0001) higher axillary shoot length growth (15.0 ± 0.41 mm) than in all the other cytokinins at various concentrations (Figure 4). In addition, 0.5 mg L^−1^ of BAP, 1.0 mg L^−1^ of TDZ, and 0.5 mg L^−1^ of KN also significantly (*p* < 0.001) increased axillary shoot length growth compared to the other treatments. The lowest mean shoot length (3.0 ± 0.24 mm) was recorded in the medium supplemented with 1.5 mg L^−1^ of 2iP. Considering the above results, 1.0 mg L^−1^ BAP supported the optimum *P. africana* axillary shoot initiation and growth in this experiment.

### Root Initiation and Growth

**Figure 5:**
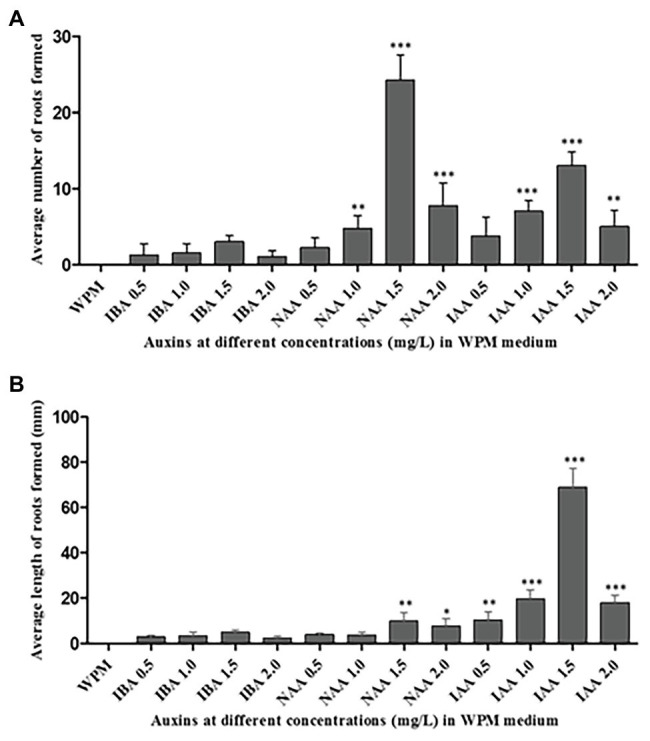
Rooting of the excised axillary shoots of *P. africana* in WPM with vitamins supplemented with 15% sucrose and different auxins at various concentrations. **(A)** Average number of the initiated roots at 6 weeks of culture. **(B)** Average length of initiated roots measured at 6 weeks of culture. The indicated statistical differences are calculated using the one-way ANOVA and Bonferroni’s *post hoc* test. ^*^ *p* < 0.05; ^**^ *p* < 0.001; and ^***^ *p* < 0.0001.

The maximum percentage of rooting was observed in axillary shoots cultured in media containing 1.5 mg L^−1^ of IAA (75% rooting), followed by 1.5 mg L^−1^ of NAA (66.70% rooting). The shoots that did not root in the first sub-culture had a 90% rooting rate in the second sub-culture. Although the highest mean number of roots (24 ± 1.65) was recorded in the medium supplemented with 1.5 mg L^−1^ NAA hormone, there was no significant difference (*p* < 0.0001) when compared to the other treatments: 2.0 mg L^−1^ of NAA, 1.5 mg L^−1^ of IAA, and 1.0 mg L^−1^ of IAA (Figure 5A). The supplementation of the medium with IBA hormone promoted very low rates of root initiation across all the concentrations tested with the highest mean root number of only 3 ± 0.41 in the medium supplemented with 1.5 mg L^−1^ of IBA for 6 weeks. Roots initiated in the medium supplemented with NAA hormones where held loosely to the callus at the shoot base. However, roots initiated in the medium supplemented with IAA hormones were stronger and more firmly attached directly to the plant base. Unlike the root numbers, which were highest in shoots cultured in the medium supplemented with 1.5 mg L^−1^ NAA hormone, the root’s length was significantly (*p* < 0.0001) longer in medium supplemented with 1.0, 1.5, and 2.0 mgL^−1^ IAA compared to the treatment with other auxins. The highest mean root length (68.75 ± 4.23 mm) was recorded in the medium supplemented with 1.5 mg L^−1^ of IAA, followed by 1.0 mg L^−1^ of IAA (mean root length = 20 ± 2.10 mm). The lowest root growth length was observed in the medium supplemented with 2.0 mg L^−1^ of IBA (2 ± 0.41 mm; Figure 5B). Consequently, 1.5 mg L^−1^ IAA supported optimum root initiation and growth.

### Acclimatization

Forty *Prunus africana* plantlets from the optimized condition with well-developed roots after 6 weeks (Figures 1E,F) were planted in sterile horticulture soil mixed with perlite in the ratio of 2:1 in plastic pots (22 cm in diameter; Figure 1G). Thirty-nine (98%) of the plants survived after 2 months in the greenhouse (Figure 1H). The plants remained healthy at 8 months in the greenhouse (Figure 1I).

### RAPD Genetic Fidelity Assessment

**Figure 6:**
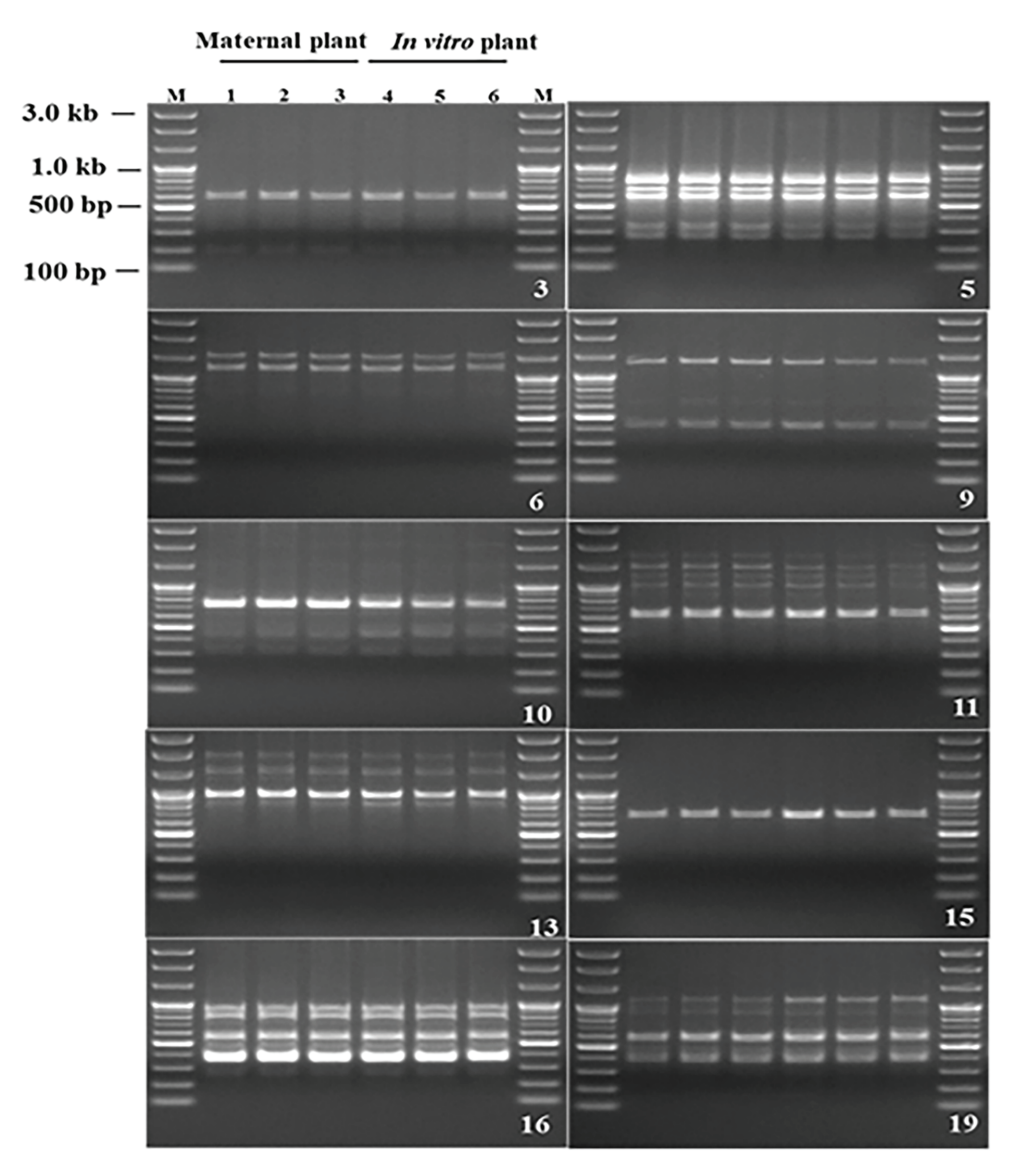
Randomly amplified polymorphic DNA profiles regenerated by PCR amplification obtained with Operon primers. Lanes M-100 by plus DNA ladder, 1–3 *P. africana* maternal plant, 4–6 *in vitro* regenerated *P. africana* plants.

Genetic fidelity analysis, using genomic DNA from both the maternal *P. africana* plant (control) and the *in vitro* regenerated *P. africana* plants, was conducted to assess genetic stability using RAPD markers. Ten RAPD primers generated 35 scorable bands in numbers ranging from 200 to 3,000 bp (Table 1). The fingerprinting profiles of the *P. africana* plants using the RAPD markers produced distinct and reproducible amplified products (Figure 6).

**Table 1:** List of primers, their sequences, number of scorable bands, and approximate sizes of the amplified fragments generated by the 10 randomly amplified polymorphic DNA (RAPD) markers.

No.	Primer code	Primer sequences(5'-3')	Number of scorable bands	Approximate range of amplification (bp)
3	OPC-04	CCGCATCTAC	1	600–700
5	OPE-04	GTGACATGCC	5	200–800
6	OPF-04	GGTGATCAGG	2	1,000–2000
9	OPI-04	CCGCCTAGTC	3	400–1,500
10	OPJ-04	CCGAACACGG	4	300–3,000
11	OPK-04	CCGCCCAAAC	6	600–1,500
13	OPM-04	GGCGGTTGTC	4	800–3,000
15	OPA-07	GAAACGGGTG	1	600–800
16	OPB-07	GGTGACGCAG	4	300–1,500
19	OPE-07	AGATGCAGCC	5	300–1,000
			Total 35	Average 510–1880

### Fourier Transform Near-IR Analysis

**Figure 7:**
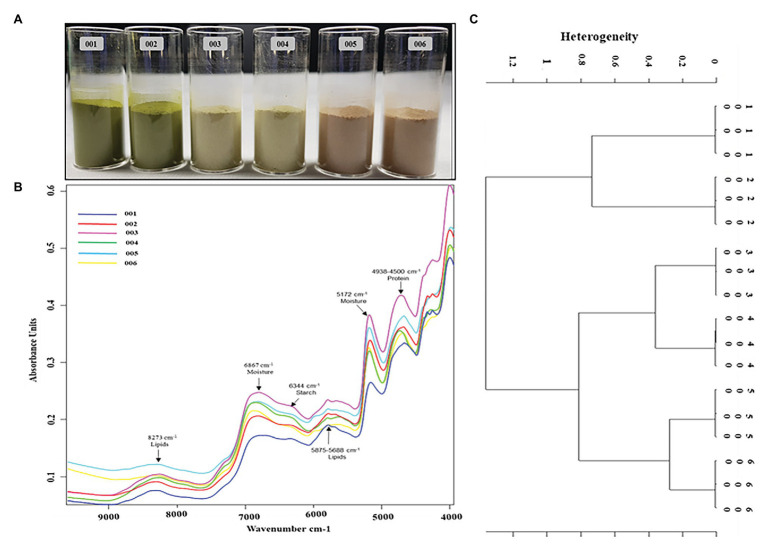
Fourier transform near-IR (FT-NIR) chemical characterization of *in vitro* regenerated and maternal *P. africana* plants. **(A)** *Prunus africana* powdered samples in 22 mm vial for FT-NIR analysis, 001 – *In vitro* regenerated plant leaf powder, 002 – Maternal plant leaf powder, 003 – *In vitro* regenerated plant stem powder, 004 – Maternal plant stem powder, 005 – *In vitro* regenerated plant root powder, and 006 – Maternal plant root powder. **(B)** FT-NIR spectra of the analyzed *P. africana* samples. **(C)** Ward’s algorithm clustering Dendrogram for the FT-NIR analyzed *P. africana* samples [Data preprocessing-First derivative + Vector normalization; Standard (Euclidean distance); Frequency ranges = 9,981–4,014 cm^−1^].

The peak assignment of the FT-NIR spectra of the analyzed *P. africana* samples 001, 002, 003, 004, 005, and 006 (Figure 7A) was done in accordance with the available literature. Six prominent FT-NIR peaks were observed around the region between 8,500 and 4,000 cm^−1^ that included 8,273, 6,867, 6,344, 5,875–5,688, 5,172, and 4,938–4,500 cm^−1^ (Figure 7B).

Fourier Transform Near-IR analysis together with vector normalization in cluster analysis reduces the variance present within a group due to the fair segregation of clusters. And based on Ward’s algorithm clustering, the vertical scale in the dendrogram (Figure 7C) represented the numerical distance between the six samples: 001, 002, 003, 004, 005, and 006. The stems and roots showed closer heterogeneity to each other than did the leaves. Hence, they were first clustered together at a distance of 0.8. The roots of the *in vitro* regenerated plants (sample 005) and those of the maternal plant (sample 006) showed the closest similarity with a heterogeneity (dissimilarity) of only 0.21. This was followed by the stems of the two plant samples (samples 003 and 004) displaying a heterogeneity of 0.28. The leaves of the two plant samples (samples 001 and 002) showed a higher heterogeneity of 0.67.

### SPAD Chlorophyll Pigment Content Assessment

There was no significant difference between the 2 year old maternal and 6 month *in vitro* propagated *P. africana* plants in terms of the SPAD values for the chlorophyll concentrations at the leaf base regions (Figure 2A), mid regions (Figure 2B), or apex regions (Figure 2C). The younger leaves of the *in vitro* regenerated and maternal *P. africana* plants measured at 1 week had lower SPAD chlorophyll contents at 48.14 ± 2.10 and 44.17 ± 3.10 values, respectively, compared with 62.96 ± 2.10 and 65.41 ± 3.10 values at 8 weeks (Figure 2D). There was no significant difference in the overall chlorophyll content distribution across the leaf surfaces.

### Measurement of Chlorophyll Fluorescence

**Figure 8:**
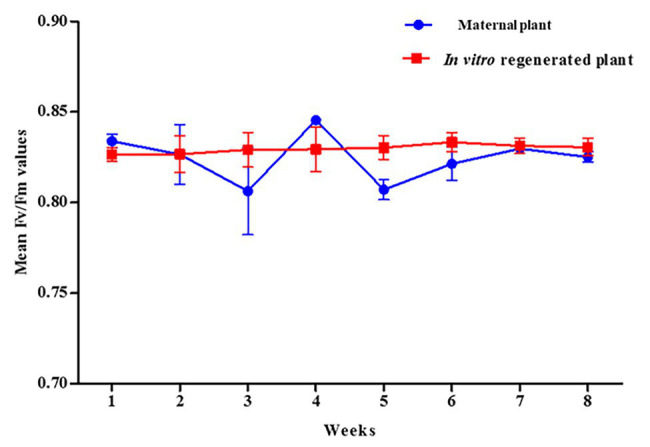
Mean Fv/Fm ratio for *in vitro* regenerated and maternal *P. africana* plants.

The potential maximum quantum efficiency of PSII photochemistry in the dark-adapted state (Fv/Fm) was measured to estimate the rate of photosynthesis in the *in vitro* regenerated and the maternal *P. africana* plants. The overall mean Fv/Fm ratio, obtained over the course of 8 weeks, for the *in vitro* regenerated and the maternal *P. africana* plants was 0.830 ± 0.0008 and 0.825 ± 0.0046, respectively (Figure 8). There was no substantial variation in the effective quantum yield of PSII between the maternal and the *in vitro* regenerated *P. africana* plants. This indicates that both plant types are likely to have similar photosynthesis rates.

## Discussion

Plant growth media play a fundamental role in plant *in vitro* propagation as it provides water, nutrients, and support to plants. Consequently, its composition can have direct effects on plant performance (Kazemia and Mohorko, 2017). As observed in this study, WPM was the optimum medium for axillary shoot initiation and growth from *P. africana* nodal segments. The suitability of WPM has also been reported for the *in vitro* propagation of *Prunus empyrean* (Sadeghi et al., 2015). The addition of sucrose to the *in vitro* culture medium provides an important source of carbon and energy for the plants (Sumaryono and Ratnadewi, 2012; Cheong and An, 2015; Elazab and Shaaban, 2015). Sucrose has also been implicated in lateral bud outgrowth (Van den Ende, 2014). As shown in this study, the axillary shoot outgrowth from the nodal segments of *P. africana* depended on the availability of sucrose in the medium. However, different concentrations of sucrose in the culture medium have been observed to exhibit different effects on the shoots of plants (Gabryszewska, 2011; João et al., 2015). This may explain the variation we found in *P. africana* nodal segment axillary shoot initiation resulting from different sucrose concentrations, with the optimum concentration found to be 15 mg L^−1^. The detrimental effect of high sucrose concentrations on the *P. africana* axillary shoot growth and elongation may be due to an osmotic imbalance, which inhibits shoot development (Karim et al., 2007).

Cytokinins are plant hormones that benefit shoot stimulation and elongation (George, 1993). In this study, 1.0 mg L^−1^ of BAP significantly (*p* < 0.0001) provided the optimum conditions for *P. africana* axillary shoot initiation from the nodal segments. The BAP hormone has previously been found to enhance *in vitro* axillary shoot initiation in a *Prunus persica* × *Prunus amygdalus* rootstock (Fotopoulos and Sotiropoulos, 2005). Indeed, the superior results of BAP in comparison to other cytokinins to the enhance shoot initiation in woody plant species was reported by Siwach and Gill (2011). This superiority may be due to the ability of BAP to trigger cell division and lateral bud development that plays a vital role in breaking axillary bud dormancy (Goyal et al., 2015; Sadeghi et al., 2015). A general decrease in the elongation of initiated *P. africana* shoots from nodal segments in the presence of high concentrations (e.g., 2.0 mg L^−1^) of the different cytokinins may be related to their vitrification effects on shoots (Kataeva et al., 1991). Previous study on *P. persica* × *P. amygdalus* rootstock also reported an inhibition of axillary shoot growth with increase in BAP concentration (Fotopoulos and Sotiropoulos, 2005).

Auxin hormones play a significant role in rooting initiation, growth, and development (Overvoorde et al., 2010; Saini et al., 2013; Sevik and Guney, 2013; Kang et al., 2018). These hormones are also important in root cell elongation and overall root growth (Pacheco-Villalobos et al., 2016). This was confirmed by our study in which the excised *P. africana* axillary shoots were rooted in WPM with vitamins supplemented with 15 g L^−1^ of sucrose and varying concentrations of auxin hormones. Previous studies have also reported that auxins enhance rooting in leafy stem cuttings of *P. africana* grown in a non-mist poly-propagator containing different growth media (Tchoundjeu et al., 2002; Kebede et al., 2013). Our results showed that 1.5 mg L^−1^ IAA significantly (*p* < 0.0001) provided the best *in vitro* rooting conditions for regenerated *P. africana* axillary shoots. The significance of IAA in adventitious root initiation and growth has also been reported in a number of previous studies (Harrison and Kaufman, 1980; Fattorini et al., 2017; Kang et al., 2018). The variation in the *P. africana* root elongation in the presence of the same hormone may result from concentration-dependent cell elongation effects (Velasquez et al., 2016). Additionally, the decrease in the *P. africana* root initiation and elongation in the presence of auxin hormones at 2 mg L^−1^ concentrations may have been caused by inhibitory effects of these hormones at such a higher concentrations (Ivanchenko et al., 2010). The acclimatization of rooted *in vitro* produced plantlets is one of the key factors in their subsequent survival in the field (Hazarika et al., 2006). This is because *in vitro* cultured plants tend to have abnormal morphologies, anatomies, and physiologies and so need time to acclimatize before they are exposed to *ex vitro* environmental conditions (Pospíšilová et al., 1999). Therefore, high survival rate (98%) of the *in vitro* propagated *P. africana* during acclimatization in this study signified their adaptability to the greenhouse conditions.

*In vitro* propagated plants are at risk of genetic variation (Sharma et al., 2016) and hence very important to assess their genetic fidelity (Min et al., 2010). RAPD, which is based on the non-coding regions of DNA, is a vital technique for efficient evaluation of genetic homogeneity and diversity (Abdelmigid and Morsi, 2018). As observed in this study, the amplified 35 products were all monomorphic bands in micropropagated plants compared with mother *P. africana* plant. No polymorphism was detected in micropropagated plant that meant genetical identity with mother plants. Our results therefore provide clear genetic information for *in vitro* produced *P. africana* plants and showed that the genetic fidelity of the *in vitro* regenerated plants was maintained throughout the *in vitro* process. The maintenance of genetic stability and uniformity in plants is vital for its proper growth, development, and reproduction (Roy, 2014). The RAPD marker technique has also been used to assess variation in other plant species, including *Swertia chirayita* (Roxb. ex Fleming) H. Karst (Sharma et al., 2016), *Mucuna pruriens* L. (Padmesh et al., 2006), *Hystrix species* (Zhang and Zhou, 2009), *Chlorophytum borivilianum* L. (Samantaray and Maiti, 2010), *Dendrocalamus strictus* (Roxb.) nees (Goyal et al., 2015), *Picrorhiza kurroa* Royle ex Benth (Rawat et al., 2010), and *Magnolia sirindhorniae* Noot. & Chalermglin (Cui et al., 2019).

The use of *FT-NIR* spectrometry, a non-destructive chemical analysis technology, has been employed in previous studies to identify and characterize chemicals and other compounds in samples (Lebot et al., 2009; Ozaki, 2012; Tamburini et al., 2015). The FT-NIR region extends from 800 to 2,500 nm (12,500–4,000 cm^−1^) and contains information about the major bonds including C–H, O–H, and N–H (Manley, 2014). In this study, the absorption band at 8,273 cm^−1^ was due to the second overtone of C-H stretch due to lipids; the large absorbance peaks around 6,867 cm^−1^ were due to the first overtone of O-H stretching and associated to the moisture content; the small absorbance peak at 6,344 cm^−1^ was due to the first overtone of the O-H stretching associated with starches. The small peak around 5,875–5,688 cm^−1^ was due to the first overtone of C-H stretching and was associated with lipids, while the absorbance peaks between 4,938 and 4,500 cm^−1^ were due to the combination of C-H, N-H stretching, and O-H stretching and were associated with proteins (Weyer and Lo, 2002; Manley, 2014; De Girolamo et al., 2019). Therefore, the similarity in the peaks of these samples associated with specific functional groups may signify the homogeneity of the *in vitro* regenerated and maternal *P. africana*. Furthermore, the spectra regenerated from FT-NIR analysis of all six samples were clustered using Ward’s algorithm. The Ward error sum of squares hierarchical clustering method has been widely used to characterize samples of this kind (Murtagh and Legendre, 2014). Indeed, in Ward’s algorithm clustering, the fusion levels are no longer spectral distances but instead illustrate heterogeneity or increasing dissimilarity in relation to the spectra formed within the clusters (Srivastava et al., 2018). Therefore, the close heterogeneity observed in the dendrogram between the respective clustered samples of *P. africana* may be attributed to the higher similarity observed between the different near-IR spectra of these respective groups of samples. This may have resulted in a lower relative distance between the samples when plotted in the dendrogram. This indicates that there is an adjacent chemical phylogenetic relationship between the samples. However, plant age may influence chemical composition, which has previously been observed in *Eucalyptus globulus* Labill (Rencoret et al., 2011), *Agave salmiana* var. ferox (Pinos-Rodríguez et al., 2008), and *Nerium oleander* L. (Achakzai et al., 2009). Therefore, plant age may explain the observed heterogeneity between clusters from the 5-month-old *in vitro* regenerated and the 1-year-old maternal *P. africana* samples. Previous studies on rice grain varieties using Ward’s algorithm for cluster analysis have reported similar findings (Srivastava et al., 2018).

Chlorophyll pigment level is one of the main indexes used to determine leaf photosynthetic ability, general plant health, and growth status (Zhang, et al., 2012; Jiang, et al., 2017). Recently, the SPAD-502 meter has been widely used to rapidly and accurately measure chlorophyll contents (Coste, et al., 2010; Ling, et al., 2011). Previous studies have observed that SPAD meter chlorophyll values are positively correlated with destructive chlorophyll measurements in a number of plants, including *Oryza sativa* L., *Glycine max* (L.) Merr., *Triticum aestivum* L. (Rodriguez, 2000), and *Solanum lycopersicum* L. leaves (Coste, et al., 2010). We observed a non-uniform distribution of chlorophyll contents across the *in vitro* regenerated and maternal *P. africana* leaf surfaces, where chlorophyll contents decreased from the leaf base to the leaf apex. This finding is consistent with previous studies on other plant species, including *T. aestivum* L. (Uddling, et al., 2007), potato (Sun, et al., 2018), and *Zea mays* L. (Moulin, et al., 2003). The observed increase in the chlorophyll contents over the 8-week experiment and eventual stability at the later stages in both the *in vitro* regenerated and maternal *P. africana* plants may have occurred because mature leaves tend to have greater and more stable chlorophyll contents than younger leaves. In other plant species, including *Vitis vinifera* L. cv. Pinotnoir (Bertamini and Nedunchezhian, 2002), *Mangifera indica* L, *Hibiscus rosa-sinensis* L., and *Psidium guajava* L. (Kamble, et al., 2015), mature leaves had higher chlorophyll contents than younger leaves. Similarities in the SPAD value readings observed in both maternal and *in vitro* propagated *P. africana* plants may indicate similarities in their rates of photosynthesis (Kura-Hotta, et al., 1987) and nitrogen content (Xiong, et al., 2015). These factors are vital for providing nutrients and signals for optimal plant growth and development (Crawford, 1995). Indeed, photosynthesis plays a major role in the energy metabolism of plants (Tanaka and Makino, 2009) and involves the conversion of absorbed light energy into chemical energy *via* the PSII and photosystem I (PSI) stages (Moustaka, et al., 2018). In the PSII stage, water is oxidized by a specialized protein complex into molecular oxygen and reduced plastoquinone, which is released into the hydrophobic core of the photosynthetic membrane. The dark-adapted values of Fv/Fm reflect the potential quantum efficiency of PSII recorded for *in vitro* regenerated and maternal *P. africana* plants in our study. As previously reported, the dark-adapted values of Fv/Fm can be used as a sensitive indicator of plant photosynthetic performance (Maxwell and Johnson, 2000; Murchie and Lawson, 2013). Therefore, the observed Fv/Fm mean values of 0.830 ± 0.0008 and 0.825 ± 0.005 for *in vitro* regenerated and maternal *P. africana* plants, respectively, at 8 weeks of experiment indicated efficiency in their photosynthetic performance and are similar to the Fv/Fm value of 0.830 reported for other plant species (Maxwell and Johnson, 2000; Murchie and Lawson, 2013). It is thought that Fv/Fm values lower than 0.830 indicate photo-inhibition (Maxwell and Johnson, 2000; Murchie and Lawson, 2013). The lack of variation in the Fv/Fm values between the *in vitro* regenerated and maternal *P. africana* plants indicate that their rates of photosynthesis are closely correlated. Chlorophyll fluorescence measurements in PSII have also been used in previous studies to assess the rate of photosynthesis in *Triticum turgidum* L., *Triticosecale witmark* cv. *“Dada”* (Moustaka, et al., 2018), and *Pseudotsuga menziesii* var. *menziesii* (Douglas fir) seedlings (Perks, et al., 2001). Thus, chlorophyll fluorescence measurements are reliable methods used to understand plant physiology based on the PSII photosynthetic process.

In conclusion, the present study describes a standard protocol for the mass propagation of an endangered medicinal plant species, *P. africana*, from stem nodal segments. WPM containing vitamins supplemented with 15 g L^−1^ sucrose and 1.0 mg L^−1^ BAP supported the optimum rate (100%) of axillary shoot initiation and growth at 6 weeks growth. WPM containing vitamins supplemented with 15 g L^−1^ sucrose, and 1.5 mg L^−1^ IAA provided the optimum rate (75%) of *in vitro* root initiation and elongation at 6 weeks growth. The *in vitro* propagated plants acclimatized well with a 98.0% survival rate. The production of monomorphic bands by the maternal plants and the *in vitro* regenerated plantlets, observed using RAPD primers, confirmed the genetic fidelity of the *in vitro* regenerated *P. africana* plants. The observed similarity in the physiological and chemical compositions between the *in vitro* regenerated and maternal *P. africana* plants further confirms that our protocol is suitable for large-scale *in vitro* propagation of genetically and physiologically stable *P. africana* plants. In this way, the future of *P. africana*, given the increasing demand for it in the herbal industry and its endangered status, may be secured. However, we recommend that additional studies on *in vitro P. africana* shoots proliferation should be conducted in the future to further add to the efficiency of production of this noble medicinal plant.

## Data Availability Statement

The raw data supporting the conclusions of this manuscript will be made available by the authors, without undue reservation.

## Author Contributions

RK conceived the original research plans, designed and conducted the research, and took part in all the stages of this study and wrote the article. Y-GK performed all the statistical analysis and wrote the manuscript. WJK performed the RAPD experiments. FO collected the plant materials that was used in this present study and wrote the article. SY authenticated the collected plant materials and gave it the herbarium code. BM performed the RAPD experiments. DO and ER performed the SPAD experiments. MM and GK revised and completed the article writing. YK technically supervised all the experiments and is the corresponding author. All authors contributed to the article and approved the submitted version.

### Conflict of Interest

The authors declare that the research was conducted in the absence of any commercial or financial relationships that could be construed as a potential conflict of interest.

## Abbreviations

RAPD: Randomly amplified polymorphic DNA
FT-NIR: Fourier transform near-IR
SPAD: Soil-plant analysis development
